# Patients’ satisfaction in positron emission tomography/computed tomography departments in Gauteng province, South Africa

**DOI:** 10.4102/hsag.v31i0.3280

**Published:** 2026-08-19

**Authors:** Themba Tshabalala, Padidi Louisa Mokoena, Lucky Chipeya

**Affiliations:** 1 Department of Medical Imaging and Radiation Sciences, Faculty of Health Sciences, University of Johannesburg, Johannesburg, South Africa

**Keywords:** patient satisfaction, positron emission tomography/computed tomography, nuclear medicine, patient experience, healthcare quality

## Abstract

**Background:**

Patient satisfaction is a subjective metric, yet an important one used for evaluating healthcare performance and identifying areas for improvement. Since the introduction of positron emission tomography/computed tomography (PET/CT) in South Africa in 2005, limited information is available on patient satisfaction in PET/CT departments. Understanding patients’ experiences in this setting is essential to ensure the delivery of high-quality, patient-centred care.

**Aim:**

This study assessed patients’ satisfaction with care when undergoing PET/CT examinations.

**Setting:**

The study was conducted at three selected academic public hospitals in the Gauteng province of South Africa.

**Methods:**

This study employed a quantitative, cross-sectional, descriptive research design to collect data from 226 randomly selected respondents between March 2022 and September 2022. A structured, closed-ended, adapted questionnaire utilising a 5-point Likert scale was used to collect data through telephone surveys.

**Results:**

This study had a predominance of female respondents (62.4%) over male respondents (37.6%). Overall, the study reported high satisfaction levels regarding staff’s professionalism (92.5%), the privacy provided in the examination rooms (94.2%), the cleanliness of the examination rooms (88.9%), reception areas (90.3%), and toilets (93.8%), while only 0.4% expressed no satisfaction at all because their appointments were not kept at the scheduled times.

**Conclusion:**

This study suggests that the majority of patients were satisfied with the level of service and care they received during PET/CT examinations. However, there is a need to improve patient appointment adherence for achieving better clinical outcomes.

**Contribution:**

Positron emission tomography/computed tomography staff should advise patients with appointment choices and provide service at the scheduled times.

## Introduction

Nuclear medicine molecular imaging (NMMI) has experienced substantial advancements over the past decade, driven by innovations in hardware and software. These developments have expanded clinical applications, particularly through hybrid modalities such as positron emission tomography/computed tomography (PET/CT) and theranostics (Vaz et al. 2020). Positron emission tomography/computed tomography integrates a PET camera and a CT scanner into a single system, enabling simultaneous acquisition of functional and anatomical data in a single examination (Doruyter et al. 2021). This hybrid modality is widely utilised in oncology for accurate tumour detection, staging, restaging and prognostication, thereby facilitating critical clinical decision-making and patient management (Hussain, Abbas & Khan 2024). Additionally, PET/CT has demonstrated increasing utility in other clinical fields, including infectious diseases, rheumatology, and cardiology, underscoring its expanding role in clinical practice (Doruyter et al. 2021).

Positron emission tomography/computed tomography imaging requires strict patient adherence to procedural instructions and efficient appointment scheduling, owing to the short half-life of radiopharmaceuticals used for imaging. Positron emission tomography/computed tomography imaging procedures can cause emotional discomfort in patients, emphasising the importance of professionals providing reassurance and a supportive environment (Sauerbrunn et al. 2026). Constrictive spaces, fear of needles, and uncertainty about examination findings can all cause patient anxiety, while long acquisition periods can exacerbate discomfort (Abreu et al. 2017; Andersson et al. 2016). Furthermore, the quality of the patient experience primarily depends on radiographers’ conduct, with empathic care improving experiences and abruptness hindering satisfaction (Munn et al. 2015).

Patient satisfaction is crucial as it provides valuable information for identifying and evaluating the quality of care delivered by healthcare professionals (Ekaterina et al. 2017). Furthermore, patient satisfaction provides feedback that can enhance the quality-of-service delivery in the healthcare sector (Ahmed, Zamzam & Hasana 2019).

Research indicates that higher levels of patient satisfaction are associated with increased patient retention, more recommendations of services to others, and improved patient adherence to health professionals’ instructions and advice (Al-Damen 2017).

The patient’s experience within a hospital setting is influenced by multiple factors encountered during interactions with various healthcare professionals and institutional services (Ahmed et al. 2019). Patient satisfaction is largely determined by two key dimensions. Firstly, it relates to patient-specific characteristics, including socio-demographic and economic factors, individual expectations, and the frequency of healthcare facility utilisation (Batbaatar et al. 2017). Secondly, the dimension encompasses provider-related factors, such as the quality of interpersonal communication, technical competence, the facility’s physical environment, and the overall accessibility of services (Batbaatar et al. 2017). The existing body of literature highlights that patient satisfaction is closely associated with demographic and sociological variables, the structural quality of healthcare institutions, the effectiveness of communication, environmental comfort, patient–provider relationships, and the technical skills of healthcare practitioners (Ahmed et al. 2019; Thornton et al. 2017).

Several studies have explored patient satisfaction across various medical fields, including nuclear medicine (Ahmed et al. 2019), radiology departments (Wahed & Mabrook 2017), and outpatient healthcare centres (Thornton et al. 2017). However, there is a lack of research on patient satisfaction in PET/CT departments since their introduction in South Africa in 2005, first in private hospitals and subsequently in public hospitals in 2007. Therefore, the aim of this study by Tshabalala (2023) was to assess patient satisfaction among individuals who underwent PET/CT examinations at selected public academic hospitals in the Gauteng province of South Africa.

## Research methods and design

### Study design

A quantitative, cross-sectional, and descriptive research design was employed for this study. The quantitative design enabled the researcher to determine factors associated with the extent of patients’ satisfaction within the PET/CT department in the Gauteng province, South Africa. Descriptive research outlined the factors affecting patient satisfaction. Cross-sectional studies are commonly used in clinical research to determine the prevalence of diseases, characteristics and health-related attitudes or behaviours (Wang & Cheng 2020:65). This design provides a ‘snapshot’ of respondents by collecting data on exposures and outcomes concurrently, with no follow-up (Wang & Cheng 2020:65). Given these characteristics, a cross-sectional approach was chosen to assess patient satisfaction with PET/CT scans at a specific moment in time. Data collection was conducted after respondents had left the department, ensuring that responses were gathered independently of PET/CT staff, thereby promoting honest, unbiased feedback.

### Study setting

South Africa’s nuclear medicine infrastructure currently comprises approximately 59 operational facilities, with 46 located in private hospitals and 13 in public hospitals. Within these facilities, over 21 PET/CT scanners are available, eight of which are located in public hospitals. As shown in Table 1, the distribution of PET/CT scanners across public hospitals varies considerably by province. Gauteng was selected as the study site because, unlike most provinces, which have few or no in-house PET/CT scanners, Gauteng serves as the national hub, with three PET/CT departments in public hospitals. The presence of multiple public hospitals equipped with PET/CT scanners in Gauteng province provided access to a substantial patient population. To maintain anonymity, the three public hospitals are referred to as Academic Hospital A, Academic Hospital B, and Academic Hospital C. Data collection was conducted consecutively across the three public hospitals, beginning with Academic Hospital A, followed by Academic Hospital B and Academic Hospital C in the subsequent months.

**TABLE 1:** Provincial distribution of positron emission tomography/computed tomography scanners in public hospitals of South Africa.

Province	Number of PET/CT facilities in public hospitals
Eastern Cape	0
Free State	1
Gauteng	3
KwaZulu-Natal	1
Limpopo	1
Mpumalanga	0
Northern Cape	0
North West	0
Western Cape	2

**Total**	**8**

PET/CT, positron emission tomography/computed tomography.

### Study population and sampling

Between March 2022 and September 2022, 226 patients were randomly selected to participate, all of whom had undergone PET/CT examinations either as initial or follow-up procedures. Eligibility was restricted to patients aged 18 years or older who had telephone access and consented verbally to participate. Patients were excluded if they were younger than 18 years, severely ill, mentally unstable, or unwilling to provide verbal consent.

The study employed simple random sampling using the fishbowl method to ensure equal selection probability for all eligible respondents, where each PET/CT department received *Protection of Personal Information Act (POPIA)* declaration forms, which served as the recruitment list, providing access to the telephone numbers as well as the ages of eligible patients following their PET/CT examinations. Each eligible patient was assigned a unique number linked to their telephone, placed into identical folded ping-pong balls, and drawn randomly every 2 weeks until the required sample size was reached.

The recommended target sample size was determined to be 360 using Taro Yamane’s formula, based on 3048 PET/CT examinations conducted across three public academic hospitals within a 1-year period, with a 95% confidence interval, a 5% margin of error, and adjustments for potential non-response error (A.G. Kuhudzai [University of Johannesburg] pers. Comm., 2021).

However, during data collection, the following obstacles were encountered: (1) rotating work-shift schedules among PET/CT staff, which led to inconsistencies in distributing information letters to potential respondents and failing to record patient details on the (*Protection of Personal Information Act* [*POPIA*]) declaration form. To address this, the researcher conducted weekly follow-ups with the PET/CT staff. (2) Technical difficulties also arose, including PET/CT scanner breakdowns and radiopharmaceutical production failures that halted PET/CT examinations from being conducted. (3) Patient recruitment posed challenges, as one patient declined participation, and two patients discontinued surveys, as they found them to be lengthy. Respondents retained the right to withdraw at any stage, in line with ethical considerations. These issues delayed data collection and prevented attainment of the recommended sample size. Nonetheless, responses from 226 respondents provided sufficient data to address the research question.

### Data collection

Data were collected using a structured, closed-ended questionnaire adapted from previously validated patient satisfaction instruments (Ahmed et al. 2019; Wahed & Mabrook 2017; Yeddes et al. 2022) used in radiology and nuclear medicine settings and modified to reflect the workflow, environment, and patient interactions specific to PET/CT imaging. The questionnaire comprised a total of 25 items, distributed across four domains, with each domain containing 5 questions. The domains were as follows:

- *Socio-demographic characteristics* – designed to collect respondents’ background information, including age, gender, educational attainment, occupation, and prior experience with PET/CT scans.
- *Service accessibility* – focused on evaluating appointment scheduling procedures, waiting times, and the clarity of pre-scan instructions.
- *Interpersonal interactions* – aimed at assessing the quality of communication, professionalism, and courtesy demonstrated by staff members, including radiographers, nurses, and administrative personnel.
- *Physical environment* – concerned with aspects such as cleanliness, privacy, comfort, and the ease of locating the PET/CT department within the hospital.

Responses in sections 2 and 3 were measured using a 5-point Likert scale, ranging from ‘not at all satisfied’ to ‘extremely satisfied’, to quantify levels of satisfaction.

Prior to commencing the study, permission was sought from the relevant hospital research committees, the heads of the PET/CT departments at the selected public hospitals in Gauteng province, and the Higher Degrees and Research Ethics Committees at the study sites. To begin with, the researcher printed and distributed sealed information letters to the PET/CT departments. On the day of their examinations, eligible patients received these letters from the PET/CT department staff after completing their PET/CT examinations. These letters outlined the objectives, procedures, advantages, and absence of risks associated with the study, along with the researcher’s contact information; patients were asked to sign a *POPIA* declaration form. By signing the *POPIA* declaration form, the respondent consented to the researcher’s access to the information specified therein, including the patient’s age, telephone number, and signature. The staff of the PET/CT department (i.e. radiographers and nurses) were exclusively tasked with providing patients with information letters and overseeing the signing of the *POPIA* declaration forms. The researcher collected these forms biweekly to conduct the surveys.

Respondents were contacted telephonically to schedule individual surveys. On the day of the scheduled survey, verbal consent was obtained from each respondent. Participation was strictly voluntary and anonymous, and respondents were assured that their responses would not affect the care they received.

During the surveys, the researcher read each questionnaire item aloud, and respondents were asked to select the number that best reflected their satisfaction level. The rating scale was explained as needed to ensure clarity and comprehension. Surveys lasted between 20 min and 35 min, and respondents were informed of the expected duration in advance. All surveys were conducted in English and were audio-recorded with respondents’ consent. The survey recordings were retained by the researcher for audit purposes and securely stored for a period of 3 years. Data collection took place from March 2022 to September 2022.

### Data analysis

Quantitative data were collated in a Microsoft Excel spreadsheet (Microsoft Corp, Redmond, WA, United States [US]) and later exported to the 2023 Statistical Package for the Social Sciences (SPSS) software, version 29 (IBM, Armonk, NY, US) for performing statistical data analysis. Descriptive statistics were used to summarise socio-demographic characteristics and the responses to satisfaction items.

These included frequencies, percentages, means, and standard deviations, as appropriate for the level of measurement. Likert scale responses were treated as ordinal data, and satisfaction scores were reported per domain (e.g. staff communication, waiting time, comfort).

### Validity and reliability

Prior to the main study, a pilot study was conducted between December 2021 and February 2022, involving 30 respondents who had previously undergone PET/CT examinations at one of the participating academic public hospitals. Respondents were selected using the same recruitment and data collection procedures intended for the main study. Based on respondent feedback, minor revisions were made to improve the tool.

The questionnaire demonstrated acceptable reliability, with a Cronbach’s alpha (α) score of 0.77, exceeding the minimum threshold of 0.70, which is generally considered indicative of internal consistency (Heale & Twycross 2015). The questionnaire was also subjected to an internal consistency test using Cronbach’s α, with an average reliability score of 0.77, which falls within the acceptable range, and additionally, to ensure rational consistency.

Face validity and content validity were confirmed during the pilot study. Face validity was established through expert review by supervisors and a statistician, who evaluated the questionnaire’s clarity, structure, and relevance, resulting in revisions that enhanced comprehension and alignment with the study objectives. Continuous input from supervisors and a statistician, together with pilot testing and institutional approval, further refined and validated the questionnaire.

To assess construct validity, a literature review conducted by the researcher identified critical factors that influence patient satisfaction, arising from a combination of demographic and sociological variables, effective communication, environmental comfort and infrastructure, patient–provider relationships, as well as healthcare provider skills. These factors guided the modification of the adapted questionnaire to ensure comprehensive coverage of the construct. The questionnaire items were aligned with these domains and were further reviewed by supervisors and a statistician to assess their relevance and representativeness. This process ensured that the instrument adequately measured patient satisfaction in PET/CT departments.

### Ethical considerations

Ethical clearance to conduct this study was obtained from the Faculty of Health Sciences Research Ethics Committee of the University of Johannesburg (No. REC-1226-2021). Further approval was obtained from the relevant hospital research committees and the head of the PET/CT department where the study was conducted.

Prior to data collection, respondents were informed in writing via an informational letter and verbally during the telephone survey about the purpose of the study.

Respondents who agreed to participate in this study were asked to provide verbal consent, which was recorded at the beginning of the telephone survey. Respondents were free to withdraw or discontinue from the survey at any point during the survey. However, withdrawal of participation after the survey was not feasible because of the research’s anonymity.

## Results

### Social-demographic profile of the respondents

A total of 226 respondents participated in this study. The socio-demographic characteristics considered in this study included age, gender, education, occupation, and the total number of visits to the PET/CT department.

Table 2 indicates the distribution of respondents by socio-demographic characteristics.

**TABLE 2:** Distribution of the socio-demographic characteristics of respondents.

Characteristics	Frequency (*n*)	%
**Total**	**226**	**100**
**Age (years)**		
18–29	22	9.7
30–41	29	12.8
42–53	42	18.6
54–65	47	20.8
66–77	56	24.8
78 and above	30	13.3
**Gender**		
Female	141	62.4
Male	85	37.6
**Education**		
Limited education	74	32.7
Matriculation	137	60.6
Diploma or degree	15	6.6
Post-graduate degree	0	0.0
**Occupation**		
Unemployed	69	30.5
Student	21	9.3
Corporate and/or Civil servant	50	22.1
Entrepreneur	15	6.6
Pensioner	71	31.4

*Source:* Adapted from Tshabalala, T., 2023, ‘Patients’ satisfaction in PET/CT departments in Gauteng province, South Africa’, Master’s dissertation, University of Johannesburg, Johannesburg, South Africa

Among the respondents, 141 (62.4%) were female, and 85 (37.6%) were male (Table 2). Respondents’ ages ranged from 18 to over 78 years, with the 66–77 age group being the most represented at 24.8%. Additionally, 137 respondents (60.6%) held a matric certificate, and 71 (31.4%) were pensioners.

**FIGURE 1:**
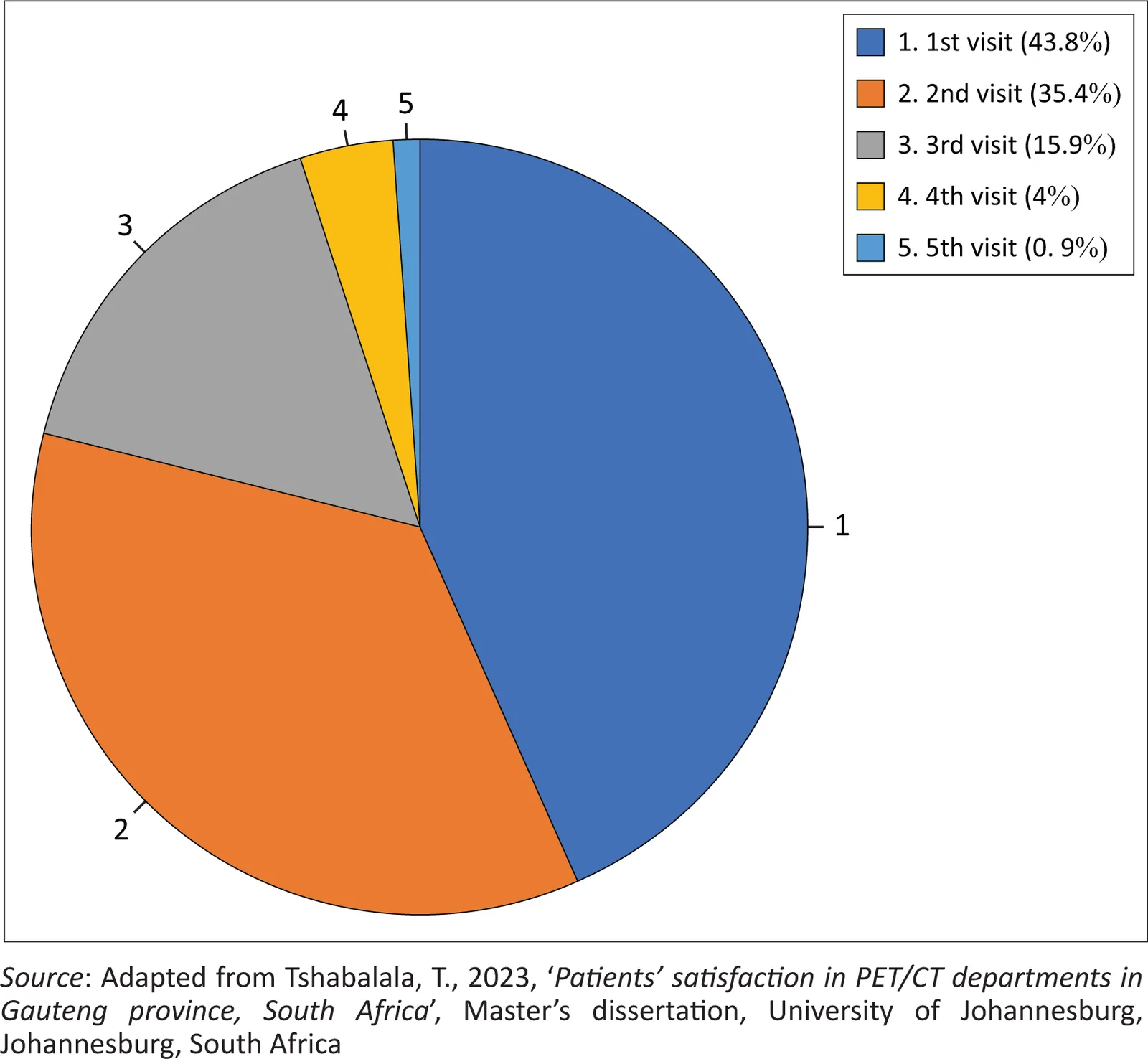
Distribution of the number of visits in the overall sample.

Each responder was asked to indicate the total number of visits to PET/CT departments; the results are shown in Figure 1.

As illustrated in Figure 1, 99 respondents (43.8%) were first-time visitors to the PET/CT department, while 80 respondents (35.4%) had visited the PET/CT department twice, representing the largest proportion of total visits recorded across all participating hospitals.

### Factors associated with the extent of patient satisfaction

#### Service accessibility

To evaluate patient satisfaction with appointment scheduling in the PET/CT scan department, respondents provided feedback on the appointment date and time, and the information received during scheduling, as presented in Table 3.

**TABLE 3:** Respondents’ rating regarding level of satisfaction with service accessibility.

Statement	Not at all satisfied		Dissatisfied		Slightly satisfied		Satisfied		Extremely satisfied		Total		Mean	s.d.
	*n*	%	*n*	%	*n*	%	*n*	%	*n*	%	*n*	%		
Choice of appointment time	0	0	0	0	31	13.7	44	19.5	151	66.8	226	100	4.53	0.725
Choice of appointment date	0	0	0	0	23	10.2	69	30.5	134	59.3	226	100	4.49	0.675
Appointment time was kept exactly as planned	1	0.4	0	0	36	15.9	35	15.5	154	68.1	226	100	4.51	0.790
Appointment date was kept exactly as planned	0	0	0	0	12	5.3	60	26.5	154	68.1	226	100	4.63	0.584
Information on what to expect on the day of the scan	0	0	0	0	5	2.2	41	18.1	180	79.6	226	100	4.77	0.469

*Source:* Adapted from Tshabalala, T., 2023, ‘Patients’ satisfaction in PET/CT departments in Gauteng province, South Africa’, Master’s dissertation, University of Johannesburg, Johannesburg, South Africa

Note: Values in this table represent the actual number of respondents (*n*).

s.d., standard deviation.

As illustrated in Table 3, regarding appointment scheduling, respondents rated the information about what to expect on scan day the highest, with an average score of 4.77. The opportunity to choose a suitable appointment date received a slightly lower score of 4.51. However, 59.3% of respondents were extremely satisfied with the option to select a convenient date, and 68.1% were extremely satisfied that their scheduled time was honoured.

#### Interpersonal interactions

Respondents’ satisfaction with PET/CT staff was assessed through questions addressing staff appearance, professionalism, and communication. Table 4 revealed important insights into the level of satisfaction with interpersonal interactions in the PET/CT setting.

**TABLE 4:** Respondents’ rating regarding level of satisfaction with interpersonal interactions.

Statement	Not at all satisfied		Dissatisfied		Slightly satisfied		Satisfied		Extremely satisfied		Total		Mean	s.d.
	*n*	%	*n*	%	*n*	%	*n*	%	*n*	%	*n*	%		
The PET/CT staff appeared neat and professional	0	0	0	0	0	0	17	7.5	209	92.5	226	100	4.92	0.264
The PET/CT staff was polite	0	0	0	0	0	0	36	15.9	190	84.1	226	100	4.84	0.367
Communication between patient and PET/CT staff	0	0	0	0	0	0	37	16.4	189	83.6	226	100	4.84	0.371
Time allocated to ask questions	0	0	0	0	0	0	41	18.1	185	81.9	226	100	4.82	0.386
PET/CT staff listened and fully address patients inquires	0	0	0	0	6	2.7	27	11.9	193	85.4	226	100	4.83	0.444

*Source:* Adapted from Tshabalala, T., 2023, ‘Patients’ satisfaction in PET/CT departments in Gauteng province, South Africa’, Master’s dissertation, University of Johannesburg, Johannesburg, South Africa

Note: Values in this table represent the actual number of respondents (*n*).

s.d., standard deviation; PET/CT, positron emission tomography/computed tomography.

As shown in Table 4, the neatness and professionalism of staff received the highest mean score of 4.92, followed by politeness and effective communication between the patient and PET/CT staff at 4.84. The time allocated for patient inquiries scored slightly lower at 4.82; however, 81.9% of respondents were extremely satisfied with this aspect.

#### Physical environment

Respondents were asked about the condition of the PET/CT department’s facility and infrastructure to assess their satisfaction with the environment. The results are outlined in Table 5.

**TABLE 5:** Respondents’ rating regarding the level of satisfaction with the physical environment.

Statement	Not at all satisfied		Dissatisfied		Slightly satisfied		Satisfied		Extremely satisfied		Total		Mean	s.d.
	*n*	%	*n*	%	*n*	%	*n*	%	*n*	%	*n*	%		
Cleanliness and tidiness of the reception area	0	0	0	0	9	4.0	13	5.8	204	90.3	226	100	4.86	0.446
Cleanliness and tidiness of the waiting room	0	0	0	0	12	5.3	18	8.0	196	86.7	226	100	4.81	0.509
Cleanliness and tidiness of the examination room	0	0	0	0	2	0.9	23	10.2	201	88.9	226	100	4.88	0.351
Cleanliness of the toilets	0	0	0	0	1	0.4	13	5.8	212	93.8	226	100	4.93	0.267
Locating PET/CT department with the hospital	0	0	0	0	11	4.9	32	14.2	183	81.0	226	100	4.76	0.530
Privacy in the examination room	0	0	0	0	0	0	13	5.8	213	94.2	226	100	4.94	0.233

*Source:* AAdapted from Tshabalala, T., 2023, ‘Patients’ satisfaction in PET/CT departments in Gauteng province, South Africa’, Master’s dissertation, University of Johannesburg, Johannesburg, South Africa

Note: Values in this table represent the actual number of respondents (*n*).

s.d., standard deviation; PET/CT, positron emission tomography/computed tomography.

The provision of privacy in the examination room received the highest mean score of 4.94, while the location had the lowest at 4.76, as shown in Table 5. A significant majority of respondents (86.7% to 90.3%) were extremely satisfied with the cleanliness of the reception area, waiting room, imaging room, and restrooms, with only a small percentage (0.4% to 4.0%) expressing slight satisfaction. Regarding the use of immobilisation devices during the PET/CT examination, room temperature, and seating availability in the waiting area, most respondents (93.4%, 79.6%, and 83.6%, respectively) were extremely satisfied, while 4.4% expressed limited satisfaction. Notably, 94.7% confirmed that their needs were met with care and empathy.

## Discussion

The demographic data for this study included respondents aged 18 to 78, with the majority falling within the 66 to 77 age group (24.8%). This suggests that older patients are more likely to access PET/CT services than younger patients in the 18–29 age group (9.7%). In contrast, a study conducted in the United Arab Emirates by Abuzaid et al. (2023) reported that the majority of respondents were between 18 and 25 years of age, whereas in a Tunisian study by Yeddes et al. (2022), most respondents were between 20 and 60 years of age. In the present study, female respondents outnumbered male respondents. These findings are consistent with those of Yeddes et al. (2022) and Al-Damen (2017), both of which reported a majority female sample. However, a study by Ahmed et al. (2019) conducted in Saudi Arabia found that the majority of respondents were male, highlighting variation in gender distribution across different geographical contexts.

With 60% of respondents in this study holding matriculation certificates, the results reflect the perspective that the majority are relatively educated individuals. These results are consistent with those from a study by Chandra, Ward, and Mohammadnezhad (2019), who found that a majority (91.5%) of respondents had completed secondary or tertiary education. Again, Owaidh et al. (2018) reported that a majority (69.6%) of the respondents in their study were either enrolled in or had completed university.

Socio-demographic characteristics in this study revealed that the majority of respondents were pensioners (31.4%) and unemployed individuals (30.5%). This is consistent with a South African study by Fernandes et al. (2020), which reported that 78.8% of the respondents were unemployed. In SA, the unemployment rate rose from 21.1% in 2007 to 29.6% in 2019 (Dadam & Viegi 2024). As a result, less than 15% of the population can afford private health insurance, leaving the majority reliant on public hospitals for healthcare (Conmy 2018; Maseko & Harris 2018).

Scheduling of an appointment is essential because it enables the Nuclear Medicine Department to provide patients with appropriate care in a timely manner, avoiding waiting periods and delays that could cause patient dissatisfaction (Akhavizadegan, Ansarifar & Jolai 2017). Patients should have the opportunity to select a convenient day and time for their radiological procedures, provided this does not impede the timely delivery of results to their referring doctors (Efanga et al. 2021). The study indicated that a significant majority of respondents were extremely satisfied with their appointment choices and the ability to keep those appointments as planned.

These results are consistent with earlier research from the United Arab Emirates (UAE), which found that patients appreciated having appointments available within a reasonable timeframe (Abuzaid et al. 2023). Nonetheless, it is important to recognise that waiting times for patients can differ significantly between various countries and healthcare settings (Makua & Khunou 2022).

Thus, Akintomide and Obasi (2019) reported that an ineffective appointment-scheduling system led to 91.82% of scheduled patients arriving late and 18.25% of them failing to show up. The reason for this was argued to be the patients’ lack of participation in the decision-making processes related to patient scheduling (Akintomide & Obasi 2019).

Moreover, a study conducted in the Radiological Department of Fayoum University Hospitals revealed that patients expressed dissatisfaction with the time spent in the waiting area, waiting for the service, and the delay between booking an appointment and the appointment date (Wahed & Mabrook 2017).

Patients have the right to understand their health conditions and treatment to make informed decisions (Perera Molligoda Arachchige & Stomeo 2024). Effective communication in Radiology and Nuclear Medicine Departments is essential for reducing procedural time, improving imaging quality, and minimising unnecessary radiation exposure (Perera Molligoda Arachchige & Stomeo 2024). This study indicated that staff in the PET/CT Department provided clear information and instructions to patients, which aligns with the findings of Abuzaid et al. (2023).

Still on effective communication and interaction, the respondents were asked whether the PET/CT staff listened to their concerns and gave them adequate time to ask questions. The study revealed that most respondents expressed extreme satisfaction with the PET/CT staff’s ability to listen to their concerns and the sufficient time allotted to address them. These findings are consistent with Iliadis et al.’s study (2022), which reported high satisfaction with nuclear medicine staff, who were willing to answer their questions.

Positive attitudes and behaviours of healthcare professionals greatly enhance patient satisfaction (Ng & Luk 2019). Patients tend to feel more satisfied when providers are friendly, courteous, and empathetic (Ng & Luk 2019). The current study indicated that most respondents were very pleased with the professionalism of the PET/CT staff. This aligns with findings from Adhikari et al. (2021), which highlight that a polite demeanour among doctors significantly contributes to high patient satisfaction.

The condition and infrastructure of a healthcare facility greatly affect patient satisfaction (Manzoor et al. 2019). This study found that most respondents were extremely satisfied with the cleanliness of the reception area, waiting room, and restrooms. Iliadis et al. (2022) also found that satisfaction with the cleanliness of the waiting area and toilets was rated high by the majority of patients. However, a study by Ekaterina et al. (2017) reported that the patients were dissatisfied with the facility’s condition. Additionally, Ahmed et al. (2019) highlighted concerns about restroom cleanliness and the insufficient number of seats in the waiting area.

Patients often experience fear and anxiety regarding medical imaging, particularly because of concerns about the examination and results, as well as fears of small spaces and needles (Grilo et al. 2020), therefore, ensuring their comfort is essential. In the current study, a high proportion of respondents expressed satisfaction with the care provided by PET/CT radiographers, who showed empathy and addressed their special needs, including the use of immobilisation devices during scans. These results are in contrast with those in a study conducted in South Africa, where patients reported dissatisfaction because of a perceived lack of attention to their needs by healthcare providers (Khumalo & Rwakaikara 2020).

Most respondents in this study reported high satisfaction with the privacy provided during their examinations. This aligns with a previous study by Odonkor et al. (2019), which also found that most patients expressed satisfaction with the privacy given during examinations. In contrast, Wahed and Mabrook (2017) reported dissatisfaction with privacy due to the inadequate design of changing rooms and examination rooms.

Overall, the study revealed that most respondents were extremely satisfied with various aspects of the PET/CT department. This aligns with the findings from the study conducted in Tunisia, where most patients expressed overall satisfaction with the quality of care in the PET/CT department (Yeddes et al. 2022); similarly, Iliadis et al. (2022) reported that almost all the patients were satisfied with their experience from visiting the nuclear medicine department in Greece.

### Limitations and recommendations

The study was limited to three selected academic hospitals in the Gauteng province, which may not fully represent the broader population of South Africa. The study’s second limitation stemmed from its quantitative research approach, which assessed patient satisfaction levels without delving further into patients’ experiences in the PET/CT department. With verbatim quotes from respondents to illustrate the degree of patient satisfaction, a qualitative research methodology would have offered a more thorough insight. Furthermore, a descriptive study cannot establish causality. Nonetheless, this study offers useful insights that can inform future research and practise about patient satisfaction in PET/CT departments in the Gauteng province, South Africa.

Given the paucity of literature on this topic in the South African context, this study makes a foundational contribution and a valuable source of preliminary data for future research.

Subsequent studies should aim to include larger, more diverse populations, encompassing a broader range of hospitals and incorporating both public and private healthcare sectors to achieve a more comprehensive and representative understanding of the South African population. Additionally, although this quantitative study provided insight into patient satisfaction levels, future research should consider adopting a mixed-methods approach to gain a deeper understanding of patients’ experiences within PET/CT departments. Such an approach would allow for both quantitative measurement and qualitative exploration of patient perceptions. Lastly, the findings of this study offer valuable insights for healthcare providers, contributing to improvements in patient satisfaction and service delivery within PET/CT departments in the Gauteng province.

## Conclusion

This study revealed that patients were generally satisfied with the level of service and care received in the three selected academic public hospitals where they underwent PET/CT examinations. However, the study also indicated a need to improve patient advice on optimal time slots and to provide services at the planned times. Consequently, in order to assess and improve the quality of care provided to patients, it is essential that patient satisfaction surveys be conducted regularly in PET/CT departments.
